# An Exploratory Analysis of Postural Stability in Acrobatic Shoulder-Stand Pyramids Using Inertial Sensors: The Interplay of Top-Athlete’s Variations and Base-Athlete’s Stance Configurations

**DOI:** 10.3390/s26185831

**Published:** 2026-09-14

**Authors:** Analina Emmanouil, Argyro Achilia, Elissavet Rousanoglou

**Affiliations:** Sports Biomechanics Lab, School of Physical Education and Sport Science, National and Kapodistrian University of Athens, 172 37 Daphne, Greece; arg.achilia97@gmail.com (A.A.); erousan@phed.uoa.gr (E.R.)

**Keywords:** biomechanics, balance routine, postural sway, inertial measurement units, stance bounding-rectangle area, postural control

## Abstract

**Highlights:**

- Base and top acceleration profiles were decoupled rather than mirrored across tiers, indicating non-uniform acceleration responses within the pyramid system.
- Top-athlete variations and base stance configurations modulate postural acceleration profiles in a directionally dependent manner, with prominent interactions in the anteroposterior-only direction.
- Within the exploratory constraints of the small sample size (five base-athletes) and confounding two top-athletes’ variations, consistent within-sample shank acceleration increases were observed in base-athletes during Pyramid T2.

**Abstract:**

In static acrobatic gymnastics pyramids, maintaining stability in a multi-person system is critical, yet unconstrained floor baselines and base-to-top pairing mechanics remain poorly quantified. This study evaluated postural stability in shoulder-stand pyramids, examining the interplay of two specific top-athlete variations (lighter Pyramid T1 vs. heavier Pyramid T2) and base-athlete stances (parallel vs. tandem). Five elite base-athletes were monitored while supporting two top-athlete variations, a lighter (Pyramid T1) and a heavier (Pyramid T2) during a standard static shoulder-stand pyramid across parallel and tandem foot placement configurations (three trials in each Pyramid variation). Unconstrained free floor-standing baselines were also recorded for base-athletes and for tops prior to and following pyramid trials. The root mean square (*RMS*) of the resultant 3D free acceleration (Xsens MTw Awinda inertial sensors sampling at 100 Hz, Xsens MT Manager version 4.6.5 software) positioned at the bases’ and the tops’ shanks (right and left) was used to assess postural stability. A five-point median filter followed by a zero-phase, 2nd-order forward and reverse Butterworth low-pass filter (yielding an effective 4th-order response at 5 Hz and 10 Hz cutoffs) was applied to all signals (MATLAB R2025b). Stance-envelope dimensions were calculated from rectangular boundaries fitted to the base’s foot outlines. Non-parametric Spearman rank correlations (ρ) and parametric correlations ($r,R^{2}$) were used to test the interbase-consistency and base-to-top coupling. Two-way repeated measures ANOVAs (pyramid x stance configurations) were applied with primary analytical emphasis placed on descriptive effect sizes alongside exact p-values (SPSS v30, *p* < 0.05). The acrobatic tandem stance expanded the parallel stance-envelope area by 148.3% and its width by 24.7%. When base-athletes transitioned from free standing to pyramids there was a substantial acceleration *RMS* increase (Pyramid T1: +24.3% to 83.6%, Pyramid T2: 47.2% to 166.2%). Supporting the heavier top-athlete (Pyramid T2) significantly increased the bases’ resultant acceleration *RMS* by +37% to +48% across both filter cutoff thresholds (p<0.05). Furthermore, top athletes exhibited differential behaviors: while Top 2 displayed lower acceleration *RMS* than Top 1, she experienced a greater acceleration surge when in Pyramid (+238.4% to +266.2%). The bases’ and the tops’ acceleration profiles did not exhibit parallel responses, indicating decoupled rather than mirrored stability adjustments. Furthermore, when the acceleration *RMS* was normalized to stance-envelope dimensions, significant pyramid x stance interaction (p<0.05) was observed in the anteroposterior but not the mediolateral acceleration. Base-athlete stability varies significantly across top-athlete variations and stance geometries. Evaluating unconstrained floor baselines alongside spatial stance boundaries is essential for capturing structural loading dynamics in multi-person athletic tasks.

## 1. Introduction

In sports science, balance ability is often considered a key predictor of athletic performance [1]. Gymnasts demonstrate superior postural control compared to non-athletes due to specialized training that enhances sensorimotor integration [2]. Acrobatic gymnastics represents an extreme manifestation of this skill, requiring a support athlete (base) and an elevated athlete (top) to function as a combined mass system (base-top pyramid pair). This pairing demands highly distinct morphological profiles, where bases typically possess greater absolute mass and structural stability, whereas tops are characterized by compact, proportional body types to facilitate balance control [3]. According to Code of Points for Acrobatics Gymnastics [4], stability is paramount during static “balance” routines, where the pyramid must be held for several seconds without visible tremors or technical faults. Research has shown that better competitive scores are directly correlated with lower center of pressure excursions (better stability) and reduced variability during pyramid execution [5,6,7].

Maintaining this stability becomes significantly more complex when external loads are introduced. The addition of an external mass—in this case, the top athlete—increases postural sway and shifts the system’s center of pressure [8,9]. Stability is further influenced by the height of the load; as its vertical distance from the base’s center of mass increases, the ability to control oscillations decreases [10,11]. This vulnerability to oscillation is heavily dictated by individual physical build, as distinct variations (morphologic somatotypes, segment distributions, and the resulting height of the center of mass) are known to alter standing posture equilibrium [12]. For the base athlete, the challenge is not only the magnitude of the top’s body mass but also the top’s individual ability to remain stable, as the total system’s stability depends on both partners [6,7]. Furthermore, the configuration of the base of support (stance configuration) is a critical determinant of stability. While a parallel foot placement provides a wider base of support, a tandem foot placement alters the stability limits in the anteroposterior and mediolateral directions [13,14]. The base’s “footedness” or preferred limb also has been reported to play a stabilizing role during asymmetrical loading [15,16].

To evaluate these complex dynamics, researchers are increasingly utilizing wearable inertial sensors. Ιnertial sensors have been successfully used to detect subtle changes in stability in specialized populations, such as ballet dancers and gymnasts [17,18]. These devices provide a valid and reliable means of assessing postural stability outside of traditional laboratory force plates [19,20,21]. Postural stability denotes the “intrinsic ability” of an individual to control subtle displacements of the line of gravity and recover from perturbations [22,23]. In the context of human movement, this control is maintained through a continuous feedback loop of visual, vestibular, and somatosensory inputs that detect segmental or whole-body accelerations [24,25].

The primary purpose of this exploratory study was to investigate postural stability in acrobatic shoulder-stand pyramids—specifically examining the interplay of top-athlete’s variations and base’s stance configuration using inertial sensors. By utilizing inertial sensors to capture real-time linear free acceleration data, this research aims to provide exploratory biomechanical information on acceleration responses during acrobatic shoulder-stand pyramids. To achieve this, the specific objectives of this exploratory study were to evaluate (a) the correlation between top and base acceleration profiles during the shoulder stand acrobatic pyramid, (b) how distinct top-athlete’s variations modulate base postural stability, and (c) whether parallel or tandem stance configurations provide directionally dependent stability for the base. Thus, this study, within the constraints of small sample size and confounding top-athlete’s variations, tested the following hypotheses:

**H1** **(Base-to-Top Coupling).** *Acceleration profiles between the top and base-athletes will exhibit non-uniform or decoupled relationships across axes, indicating independent stabilization strategies rather than a rigid, mirrored transmission of the top-athlete’s movement to the base.*

**H2** **(Top-Athlete Variations).** *Pyramids involving the higher-demand top-athlete variations will result in decreased base postural stability (higher linear accelerations) compared to the lower-demand profile.*

**H3** **(Stance Configuration).** *The effect of stance configuration (parallel vs. tandem) on the base’s postural stability will be directionally dependent, exerting differential impacts across anteroposterior and mediolateral axes.*

## 2. Materials and Methods

### 2.1. Participants

A total of seven young female athletes (age range from 7.7 to 15.7 years) participated in the study. Their physical characteristics are presented in Table 1. Five participants were competing as support athletes (bases), while two were competing as flyer athletes (tops), designated as the Top 1-T1 and Top 2–T2, corresponding to the two specific top-athlete variations examined in this exploratory study (lighter- and heavier-athlete variations). Due to training team availability, the prior joint training experience varied across base-top pairings. To minimize the acute effect of pairing unfamiliarity during testing, all pairs completed a standardized warm-up and practice protocol prior to data collection.

While the sample size of five elite base-athletes is modest in absolute terms, it reflects the inherent constraints of recruiting highly specialized, elite-level cohorts in acrobatic gymnastics. Elite acrobatic partnerships require strict anthropometric pairing, complementary skill profiles, and synchronized competitive experience, making the population of eligible and active partnerships exceptionally limited at the national competitive level.

Selection criteria included active competitive experience in official acrobatic gymnastics events and specific anthropometric profiles (particularly for the tops), as these characteristics were expected to influence the stability of the bases [3,12]. Because the heavier top athlete also differed in age and body height, the top-athlete conditions completely confound mass with other physical and skill-related variables. Consequently, comparisons between these two conditions reflect an integrated response to the overall top-athlete physical and balancing skill profile rather than an isolated effect of mass alone. Exclusion criteria included current musculoskeletal injuries or the absence of a valid athletic license from the Hellenic Gymnastics Federation. All procedures were approved by the Internal Research Ethics and Bioethics Committee of the School of Physical Education and Sport Science (Ref: 1470/11-01-2023). Written informed consent was obtained from the parents or legal guardians of all participants.

### 2.2. Anthropometric Measurements

**Figure 1:**
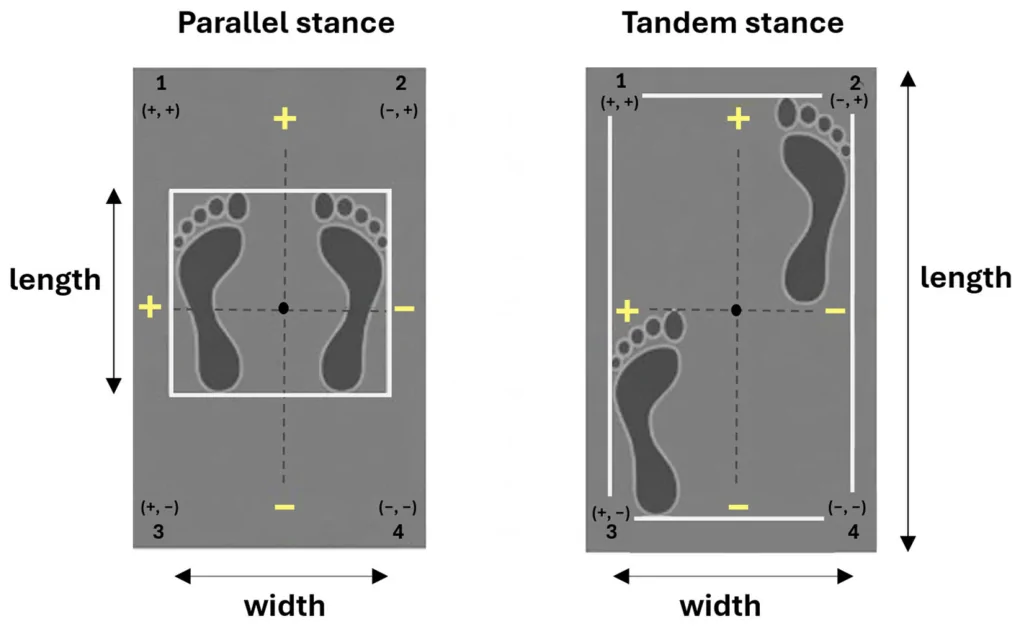
Schematic representation of foot placements in the parallel (**left**) and the tandem (**right**) stances. The white rectangle indicates the bounding rectangle used to calculate the stance-envelope area, defined as the product of its ML (width) and AP (length) dimensions.

Testing was conducted at the Sports Biomechanics Laboratory of the National and Kapodistrian University of Athens with participants wearing typical athletic attire. Body mass was measured using a precision analog scale (Seca, accuracy 0.1 kg), and body height was measured with a wall-mounted stadiometer (Seca). Participants were instructed to remain as still as possible, maintaining their head aligned with the spine and their gaze fixed on a target at eye level. To ensure consistency across trials, each athlete’s foot placement (base participant) was traced onto a fixed cardboard template for both the parallel and tandem stances (Figure 1).

### 2.3. Accelerometric Data Collection

A total of six MTw Awinda Xsens inertial sensors were used for accelerometric data collection using the Xsens MT Manager version 4.6.5 data recording software (Xsens Technologies B.V., Enschede, Overijssel, The Netherlands). Four sensors were dedicated to the two top-athletes, placed bilaterally on their right and left shanks, with each sensor remaining attached to the top-athlete throughout all trials. The remaining two sensors were attached bilaterally to the right and left shanks of the tested base-athlete and remained attached throughout the specific base’s testing session. The sensors were positioned securely at the right and left shank center of mass (CoM) approximately at 43.52% of the segment’s length measured from the knee joint center down the segment line [26] (each one sampling at a frequency of 100 Hz) and were used to record the 3D free linear acceleration (m/s^2^) provided directly by the Xsens MTmanager version 4.6.5 data collection software (Xsens Technologies B.V., Enschede, Overijssel, Netherlands). All four sensors in the Pyramid trials (and the two sensors in the free base and the free top trials) were synchronized natively using the Xsens MTw Awinda Master Station [27]. The Awinda Master serves as an interface between the Awinda host (the PC running MT Manager version 4.6.5 Xsens-based data recording software), and one or more MTw inertial sensors. It uses a proprietary, high-precision wireless protocol (Awinda protocol) to broadcast a common time-sync pulse to all connected sensors simultaneously, maintaining sub-millisecond synchronization across all active sensors throughout the recording session [27]. Anatomical calibration indicated that the *X*-axis recorded the mediolateral (ML) acceleration, the *Y*-axis the vertical (Vert) one, and the *Z*-axis the anteroposterior (AP) movement.

### 2.4. Experimental Procedure

**Figure 2:**
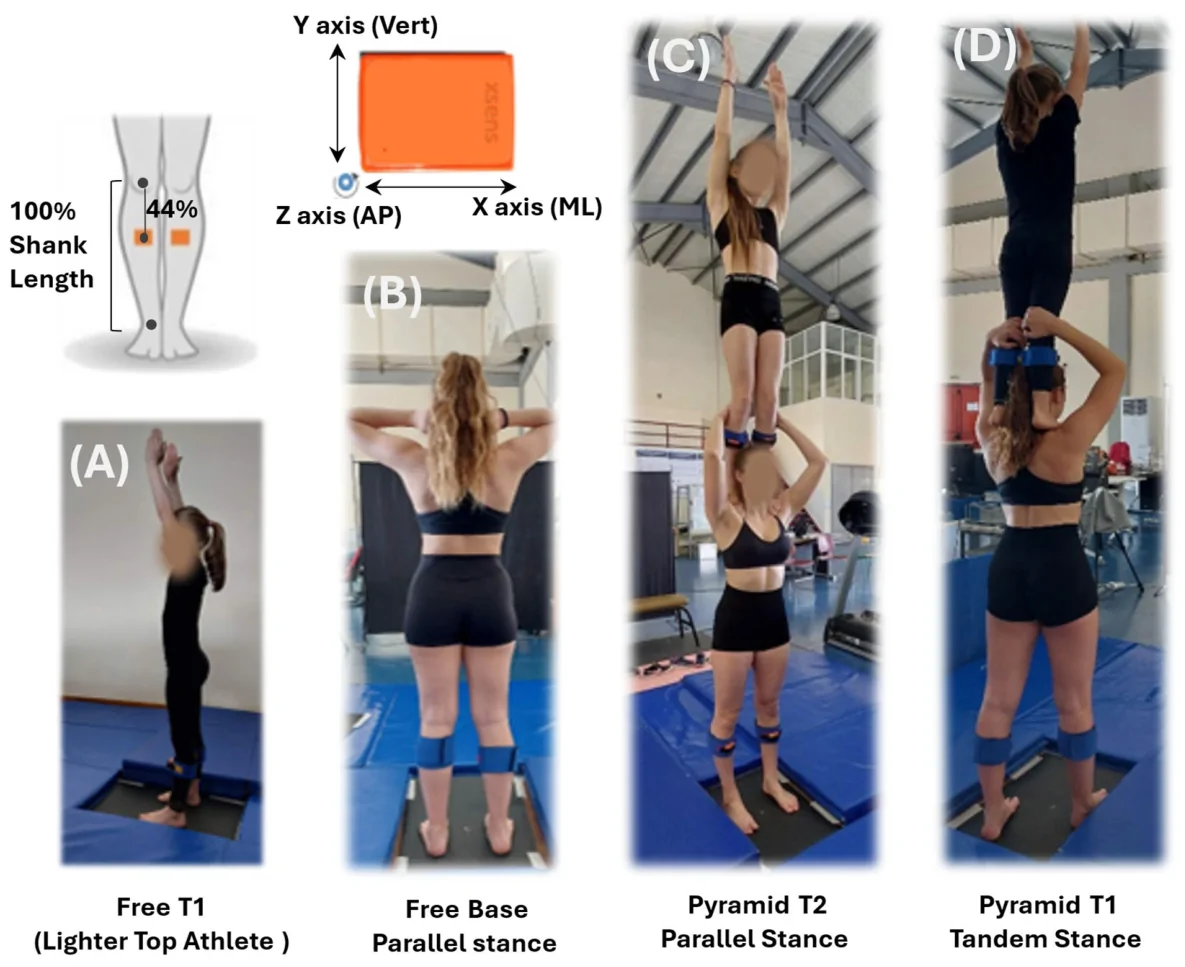
Τhe inertial sensor used in the study, its placement on the shank and the axes anatomical calibration. (**A**) free top condition, (**B**) free base condition at parallel stance, (**C**) pyramid at parallel stance and the heavier-top athlete (Pyramid T2), (**D**) pyramid at tandem stance and the lighter-top athlete (Pyramid T1). In free top and free base conditions, the athletes maintained the depicted upper arm configuration to best approximate the pyramid configuration. The blue elastic Velcro straps secured the inertial sensors at the shank.

Each base participant performed the parallel and the tandem stances (Figure 2) with both top-athlete variations, Pyramid T1 and Pyramid T2. The top-athlete variation and the stance configuration order were administered using a counterbalanced rotation, such that each successive participant commenced with the alternative stance configuration and top-athlete variations. Each trial lasted 20 s, the trial recording started after the top had stood on the base’s shoulders, a 2 min rest was provided between trials, and no trial had to be discarded due to bad performance or loss of balance. Following general familiarization with the experimental procedure, two short-duration familiarization trials (ascending to the base’s shoulders, standing for 5 s, and descending) were performed in each stance configuration.

The bases also performed three unconstrained floor standing trials without supporting any top (free base condition) and the tops three free floor standing trials (free top condition) prior (pre free top) and following (post free top) the pyramid trials as a baseline reference assessment of the individual balancing skill as well as for assessing the tops’ potential fatigue response.

As shown in Figure 1 and Table 2, the tandem stance was a staggered one, increasing by about 2.5 times the area of the bounding-rectangle stance-envelope (calculated via stance length multiplied by width) compared to the parallel, with its length being increased by 1.8 times and its width by 1.4 times. The length increase varied more (CV: 11.1%) than the width increase (CV: 7.0%).

### 2.5. Data Analysis

Data Preprocessing and Windowing. The accelerometric signal was processed using the MATLAB R2025b software (MathWorks, Natick, MA, USA). To eliminate initial experimental adjustment transients, the initial 5 s of data were excluded [28]. A subsequent analysis window of 15 s (spanning from t=5 s to t=20 s) was isolated for rigorous evaluation. To prevent edge artifacts from initialization of digital filtering, a 1 s temporal buffer was integrated prior to the primary analysis window (from *t* = 4 s) [29]. Following the filtering pipeline, this buffer was trimmed, securing a stable 15 s analysis window (5 s to 20 s), corresponding to 1500 samples at a sampling frequency 100 Hz. No linear detrending or mean-centering was performed prior to *RMS* calculations, as visual inspection confirmed that dynamic acceleration signals were centered around $0{\;m/s}^{2}$ following integrated gravity removal via the Xsens sensor fusion algorithm [27], with no evident low-frequency baseline drift across the 15 s trials. Across all raw signals, a preliminary 5-point median filter was applied to attenuate high-amplitude impulse noise and sensor spikes without distorting the underlying signal dynamics [30].

**Figure 3:**
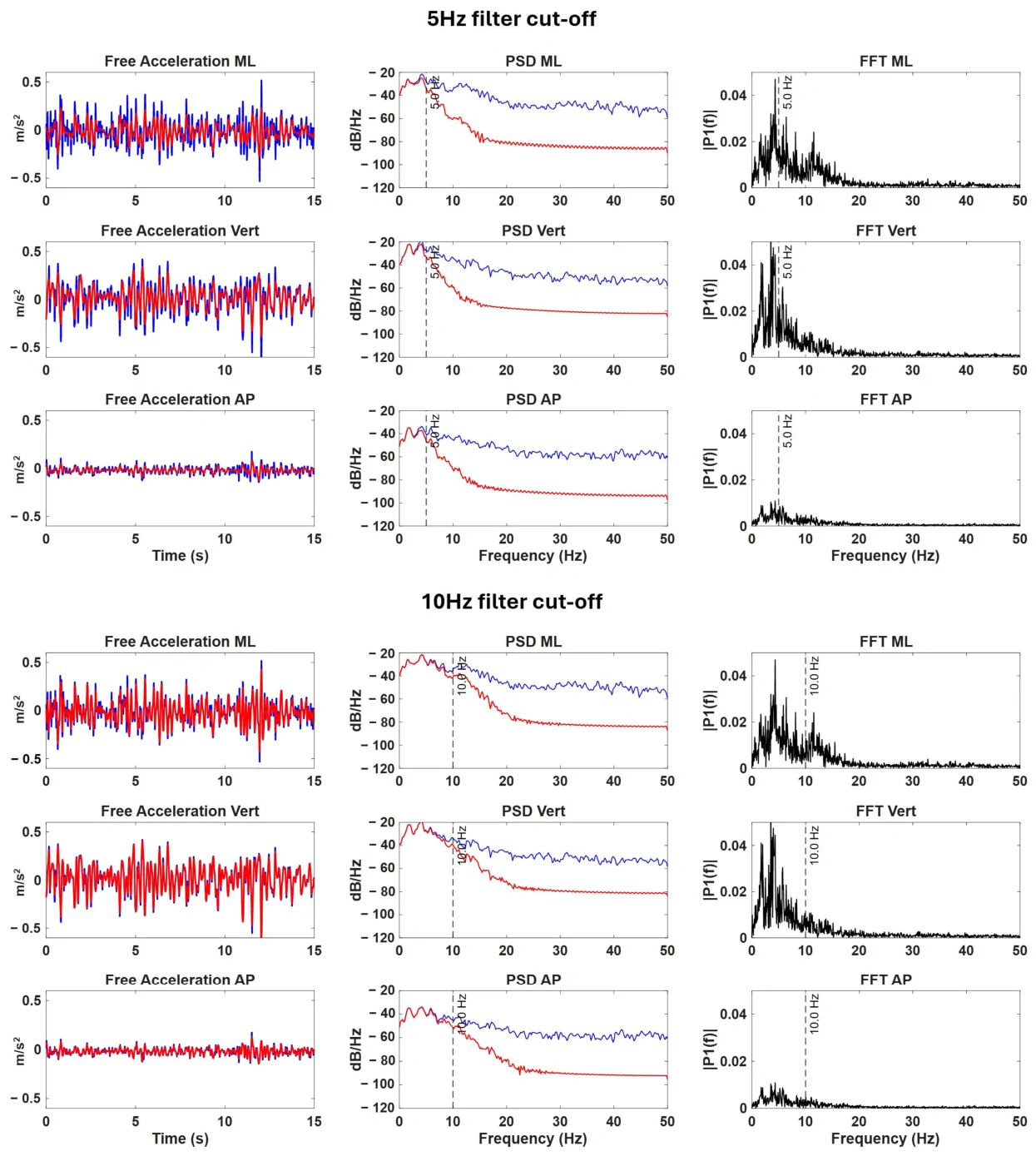
Representative signal processing workflow and frequency analysis of the free acceleration recordings during static acrobatic shoulder stand pyramid. (**Left column**): Free linear acceleration signals (${\mathrm{in}\;m/s}^{2}$) over time (s) for mediolateral (ML), vertical (Vert), and anteroposterior (AP) axes, comparing the original unfiltered (blue) against the filtered (red) signal. (**Middle column**): Power Spectral Density (PSD) in dB/Hz plotted against frequency (Hz), featuring the cutoff frequency marker (5.0 Hz or 10.0 Hz). (**Right column**): Fast Fourier Transform (FFT) amplitude spectrum ($\left|P1\left(f\right)\right|$) depicting the dominant frequency distribution up to 50 Hz.

**Figure A1:**
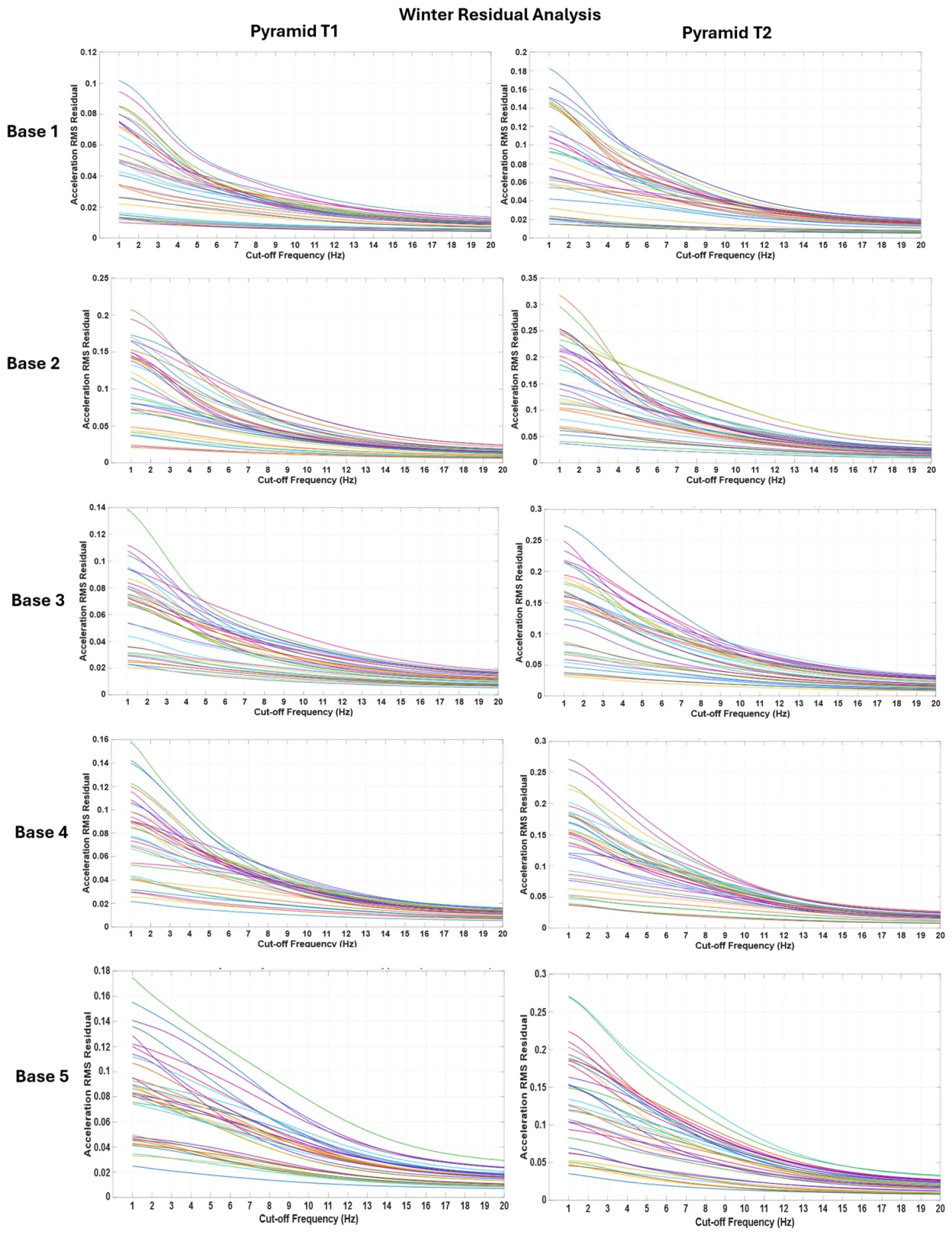
Winter’s residual analysis curves illustrating the root-mean-square (*RMS*) acceleration difference between unfiltered and low-pass filtered signals in the five base-athletes, across all acceleration signals (each line color represents a different trial). Cutoff frequencies were tested from 1 Hz to 20 Hz in 0.1 Hz increments using a 2nd-order zero-phase Butterworth filter (filtfilt). Each panel displays individual channel decay trajectories for Pyramid T1 (lighter top-athlete) and for Pyramid T2 (heavier top-athlete), demonstrating the transition from high-frequency noise removal into the asymptotic plateau. The chosen cutoff thresholds (5 Hz and 10 Hz) sit within and beyond the primary decay zone to balance noise attenuation with movement dynamics.

Filter Cutoff Optimization via Residual Analysis. Signal power distribution and frequency content were assessed using Power Spectral Density (PSD) estimation via Welch’s method (pwelch) [31] alongside Fast Fourier Transform (FFT) analysis (Figure 3). To objectively determine and justify the optimal low-pass filter frequency across the varying axes and balancing conditions, Winter’s residual analysis was implemented [28] (Winter’s residual analysis indicative plots are presented in Figure A1). Because biomechanical signals originating from different anatomical axes and sensor channels exhibit varying power distributions and non-stationary characteristics, testing a wide spectral band via residual analysis is essential. A 2nd-order Butterworth low-pass filter using a bidirectional, zero-phase forward and reverse filtering scheme (filtfilt function, [32]) was applied across a test frequency range from 1 Hz to 20 Hz with 0.1 Hz increments. The root-mean-square (*RMS*) difference (residual) between the pre-filtered and low-pass filtered signals was computed to identify the optimal threshold where high-frequency noise reaches an asymptote. The Winter’s residual analysis indicated that 5 Hz sits within the transition zone of the error curves, serving as a conservative threshold for attenuating high-frequency noise, whereas 10 Hz approaches the asymptotic plateau region, capturing faster stabilization dynamics across all channels. Consequently, both frequencies were implemented and compared to evaluate their respective effects on signal magnitude (*RMS*) [28,33].

Filtering, Spectral Analysis, and Performance Metrics. Following determination, a standardized low-pass filter configuration (utilizing a 2nd-order Butterworth design normalized to the Nyquist frequency) was applied across all data. The Butterworth topology is selected for its maximally flat magnitude response in the passband to prevent artificial amplitude distortion [34]. To eliminate time delay and phase shift, a bidirectional zero-phase filtering scheme (filtfilt [32]) was executed, by processing the signal forward and backward in time (yielding an effective 4th-order filter response), at both 5 Hz and 10 Hz frequencies, to ensure perfectly synchronized, zero-phase distortion [29]. In both frequencies, acceleration *RMS* (expressed in m/s^2^) of the left and right shank was computed for a 15 s analysis window (spanning from t=5 s to t=20 s) in the AP, ML, and Vert directions (Figure 3), during the two stance configurations in Pyramid T1 and Pyramid T2, as well as during the free base and the free top conditions.

### 2.6. Two-Metric Biomechanical Framework

The postural stability hypotheses of the study were tested employing the following two-metric biomechanical framework, (a) the resultant *RMS* acceleration, and (b) the acceleration *RMS* normalized to the dimensions (width and length) of the bounding-rectangle stance-envelope.

Resultant Acceleration *RMS*. To quantify the 3D acceleration magnitude experienced by the base (total postural stability), the resultant *RMS* values from the AP, ML and Vert axes were combined using the Pythagorean norm. This was calculated independently for the right [Equation (1)] and the left [Equation (2)] shank:

(1) $${\mathrm{Resultant}\;\mathrm{RMS}}_{\mathrm{Right}}=\sqrt{RMS_{AP_{Right}}^{2}+RMS_{ML_{Right}}^{2}+RMS_{Vert_{Right}}^{2}}$$

(2) $${\mathrm{Resultant}\;\mathrm{RMS}}_{\mathrm{Left}}=\sqrt{RMS_{AP_{Left}}^{2}+RMS_{{ML}_{Left}}^{2}+RMS_{Vert_{Left}}^{2}}$$

The resulting values for the right and left shank in each trial were subsequently averaged (resultant *RMS*_RL_) to yield a single unified *RMS* acceleration value [Equation (3)], and the three-trial average was used in the statistical analysis.

(3) $$\mathrm{Single}\;\mathrm{Trial}\;\mathrm{Resultant}\;{\mathrm{RMS}}_{\mathrm{RL}}=\frac{{\mathrm{Resultant}\;\mathrm{RMS}}_{\mathrm{Right}}+{\mathrm{Resultant}\;\mathrm{RMS}}_{\mathrm{Left}}}{2}$$

Normalized Acceleration *RMS*. To account for spatial boundary constraints associated with stance configuration, the directional acceleration *RMS* was normalized to the physical dimensions of the base’s bounding-rectangle stance-envelope. The RL_average_ AP acceleration *RMS* was normalized to the stance-envelope length, L, to obtain AP *RMS*_norm_ [Equation (4)], and the RL_average_ ML acceleration *RMS* was normalized to the stance-envelope width, W, to obtain ML *RMS*_norm_ [Equation (5)]. The stance-envelope width and length were expressed in cm. To improve readability, the normalized values were scaled by a factor of 100 and expressed as acceleration units per meter of the stance-envelope boundary (m/s^2^ per m). The three-trial average was used in the statistical analysis.

(4) $$\mathrm{AP}\;{\mathrm{RMS}}_{\mathrm{norm}}=\frac{AP\;{\mathrm{RMS}}_{RL}}{L}$$

(5) $$\mathrm{ML}\;{\mathrm{RMS}}_{\mathrm{norm}}=\frac{ML\;{\mathrm{RMS}}_{RL}}{W}$$

### 2.7. Statistical Analyses

To evaluate inter-base consistency and base-to-top mechanical coupling (H1), bivariate non-parametric Spearman rank correlation coefficients (ρ), parametric Pearson correlation coefficients (r), and coefficients of determination ($R^{2}$) were computed across stance configurations using the three-trial averaged dynamic acceleration (*RMS*) metrics. Given the small sample size (N=5) and potential departures from normality, non-parametric Spearman rank correlation coefficients (ρ) [35] were designated as the primary statistical measure to evaluate monotonic rank stability without parametric distributional assumptions, while Pearson metrics were evaluated for linear variance comparisons. Spearman correlation magnitudes were interpreted in accordance as negligible/weak (0.00–0.29), moderate (0.30–0.49), strong (0.50–0.69), very strong (0.70–0.99), and perfect (1.00) [35]. Pearson correlation effect sizes were interpreted using Cohen’s benchmarks [36] (r=0.10, small; r=0.30, medium; r=0.50, large).

To test the main and interaction effects of top-athlete’s variations (lighter vs. heavier) and foot placement (parallel vs. tandem stance) on base-athlete’s postural, two-way repeated-measures ANOVA were conducted on resultant acceleration *RMS* and directionally normalized acceleration *RMS* (AP *RMS*_norm_ and ML *RMS*_norm_). Given the exploratory nature of this study and the small sample cohort, statistical reporting emphasized p-values, confidence intervals (CI), and effect sizes (*η*^2^*_p_*) alongside Bonferroni-adjusted significance thresholds for pairwise comparisons. All statistical evaluations were cross-verified separately across 5 Hz and 10 Hz low-pass filter cutoffs. SPSS v30.0 (IBM Corp., Armonk, NY, USA) was used for all analyses, with significance threshold at α=0.05. Because the SPSS software does not natively output CI for ANOVA effect sizes, a custom MATLAB script (MATLAB R2025b software, MathWorks, Natick, MA, USA) (provided as Supplementary Material) was used for their computation.

## 3. Results

### 3.1. Base and Top Athletes’ Postural Dynamics: Free Standing vs. Pyramid Integration

**Figure A2:**
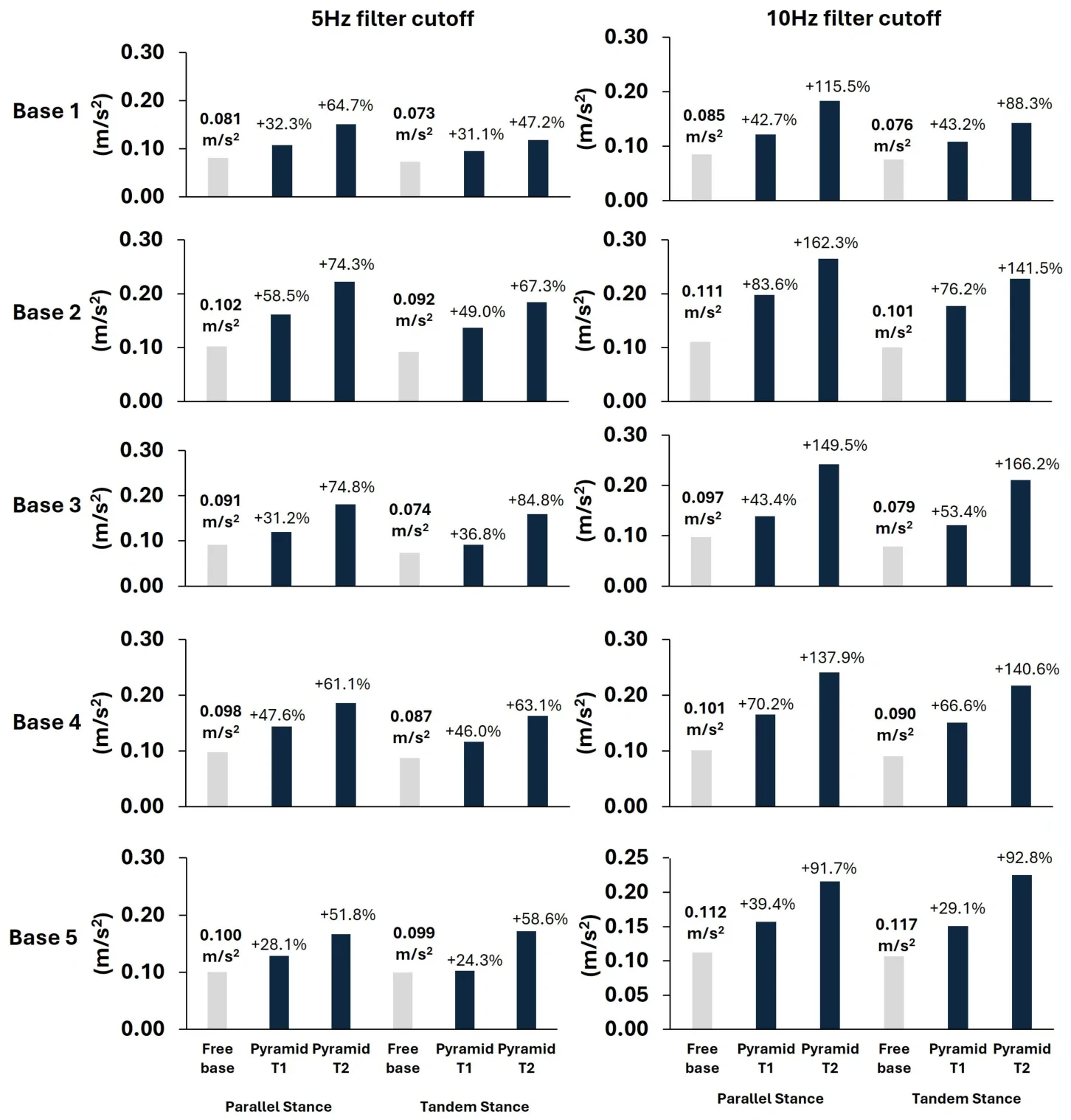
Resultant *RMS* acceleration (${m/s}^{2}$) of individual base-athletes (Base 1 to Base 5) across free floor-standing baselines (Free base) and pyramid configurations (Pyramid T1 and Pyramid T2) in parallel and tandem stances, processed at 5 Hz (**left panel**) and 10 Hz (**right panel**) low-pass filter cutoffs. Numerical values above free base bars indicate absolute baseline acceleration magnitudes (${m/s}^{2}$). Percentage values (%Δ) above Pyramid T1 and Pyramid T2 bars represent acceleration amplification relative to the respective free floor standing baseline.

Base-Athletes Postural Dynamics. During unconstrained floor standing, base-athletes exhibited baseline resultant *RMS* values ranging from 0.0163 to 0.0232 m/s^2^ at the 5 Hz cutoff and from 0.0129 to 0.0265 m/s^2^ at the 10 Hz cutoff across stance variations (see Appendix A, Figure A2 for individual profiles). Transitioning to pyramid integration significantly amplified resultant acceleration *RMS* across both parallel and tandem stance configurations at the 5 Hz and 10 Hz filter cutoffs (*p*_adj_ < 0.001, Table 3).

**Figure 4:**
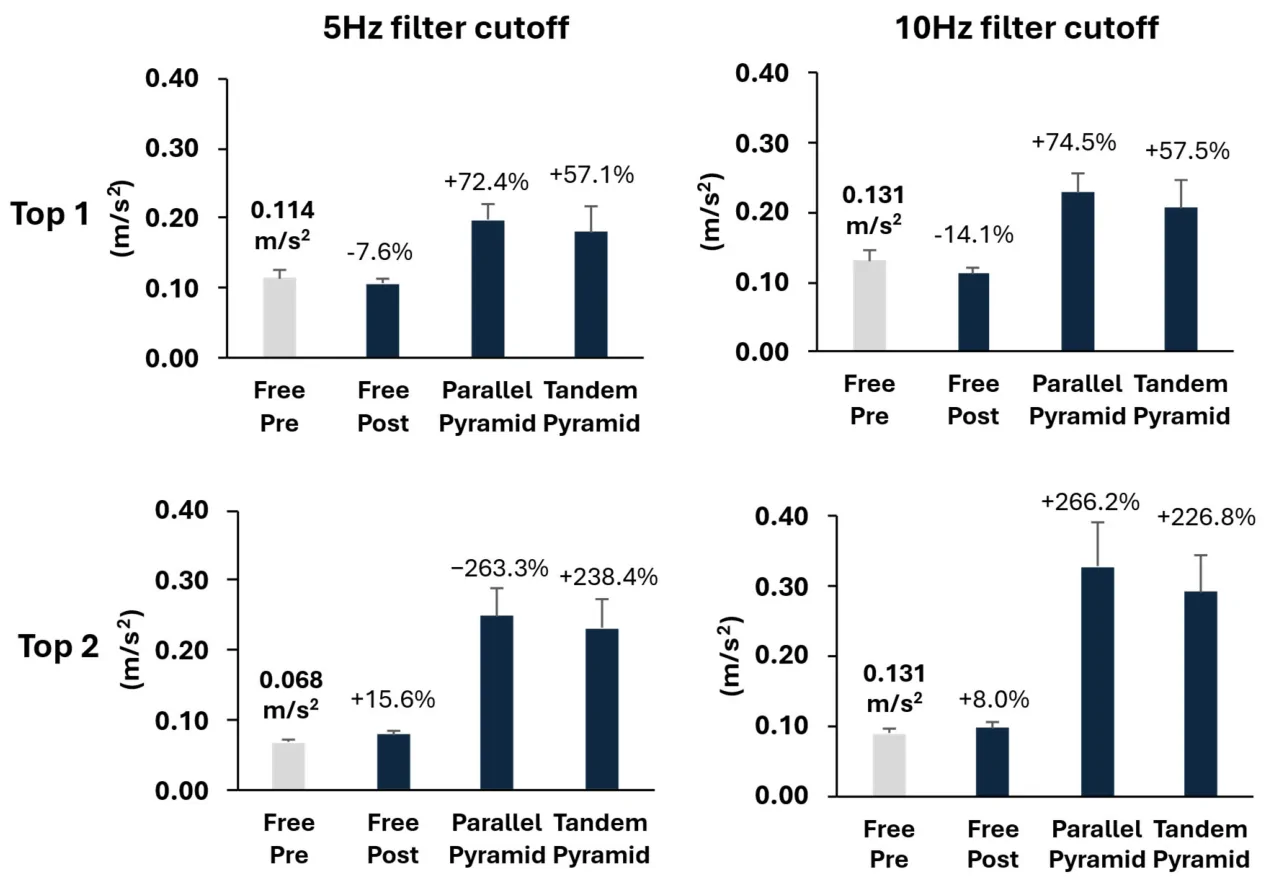
Resultant *RMS* acceleration ($m/s^{2}$) of Top 1 and Top 2 across free floor-standing baselines (Pre and Post) and pyramid configurations (Parallel and Tandem) processed using 5 Hz (**left column**) and 10 Hz (**right column**) low-pass filter cutoffs. Values represent mean and standard deviation. Percentage values above pyramid configurations represent relative change (%Δ) compared to the pre-test floor baseline.

Top-Athletes Postural Dynamics. Unconstrained floor standing established distinct baseline profiles for top-athletes, with Top 1 showing higher baseline resultant *RMS* values than Top 2, and both maintaining minimal variation across pre- and post-test trials. Upon integration into the pyramid structures, both athletes experienced substantial acceleration increases relative to their free floor standing baselines across all frequency cutoffs and stances (Figure 4). Top 2 demonstrated a more pronounced postural adjustment surge compared to Top 1 in both parallel and tandem configurations (Figure 4).

### 3.2. Inter-Base Consistency and Base-to-Top Coupling: Correlation Analysis

Across both 5 Hz and 10 Hz low-pass filter cutoffs, non-parametric Spearman rank correlation (ρ) and parametric Pearson correlation (r) analyses revealed consistent structural patterns regarding relative stability metrics and dynamic balance behavior (Table 4). Given non-normal distributions and small sample constraints (N=5), Spearman rank correlation coefficients (ρ) served as the primary measure of monotonic association, while parametric Pearson metrics ($r,R^{2}$) are provided for linear variance comparison.

Inter-Base Consistency Across Stance Configurations. Strong positive correlations were demonstrated between bases across the two pyramid variations (Base Pyramid T1 vs. Base Pyramid T2) (Table 4), suggesting that the relative ranking of base-athlete acceleration responses was largely preserved between Pyramid T1 and Pyramid T2 in both stance and cutoff conditions. In the parallel stance, primary non-parametric evaluation demonstrated strong inter-base rank consistency at the 5 Hz cutoff (ρ=0.90,p=0.037), supported by a strong linear association (r=0.92,p=0.027). At the 10 Hz cutoff, rank consistency remained moderate-to-strong (ρ=0.70,p=0.188), whereas the linear correlation retained statistical significance (r=0.89,p=0.046) (Table 4). In the tandem stance, primary non-parametric evaluation confirmed strong inter-base rank consistency at the 5 Hz cutoff (ρ=0.90,p=0.037), whereas the linear relationship reached a near-significant threshold under parametric testing (r=0.86,p=0.061). At the 10 Hz cutoff, perfect monotonic rank agreement was observed (ρ=1.00,p<0.001), though the linear Pearson association remained slightly attenuated by sample size constraints (r=0.87,p=0.058) (Table 4). This pattern allows reporting both rank-based and linear associations in this small exploratory sample.

Base-to-Top Coupling. Consistently across both 5 Hz and 10 Hz cutoffs, coupling strengths were higher in Pyramid T2 (Base T2 vs. Top 2) than in Pyramid T1 (Base T1 vs. Top 1) across parallel and tandem stances (Table 4). In the parallel stance at 5 Hz filtering, Spearman analysis indicated a significant rank-based association for Pyramid T2 (ρ=0.90,p=0.037); the corresponding Pearson correlation did not reach statistical significance (r=0.78,p=0.118). For all other base-to-top stance and cutoff conditions, both Spearman and Pearson coefficients yielded non-significant coupling (p>0.05) (Table 4).

### 3.3. Main Effects—Interaction of Pyramid and Stance Configuration on Base’s Resultant Acceleration RMS

Repeated-measures analyses revealed a significant main effect of pyramid configuration on base-athlete resultant *RMS* acceleration (*F*(1,4) = 12.943, *p* = 0.023, partial *η*^2^*_p_* = 0.764, 90% CI [0.098, 0.856] at 5 Hz; and *F*(1,4) = 11.728, *p* = 0.027, partial *η*^2^*_p_* = 0.746, 90% CI [0.083, 0.850] at 10 Hz). A significant main effect of stance configuration was also observed at both 5 Hz (*F*(1,4) = 92.910, *p* < 0.001, partial *η*^2^*_p_* = 0.959, 90% CI [0.705, 0.974]) and 10 Hz (*F*(1,4) = 82.279, *p* < 0.001, partial *η*^2^*_p_* = 0.954, 90% CI [0.675, 0.971]). However, the interaction between pyramid and stance configurations was not statistically significant at either filter frequency (5 Hz: *F*(1,4) = 1.142, *p* = 0.345, partial *η*^2^*_p_* = 0.222, 90% CI [0.000, 0.615]; 10 Hz: *F*(1,4) = 1.621, *p* = 0.269, partial *η*^2^*_p_* = 0.288, 90% CI [0.000, 0.651]), indicating that the influence of stance on postural stability was not affected by top-athlete variations.

**Figure 5:**
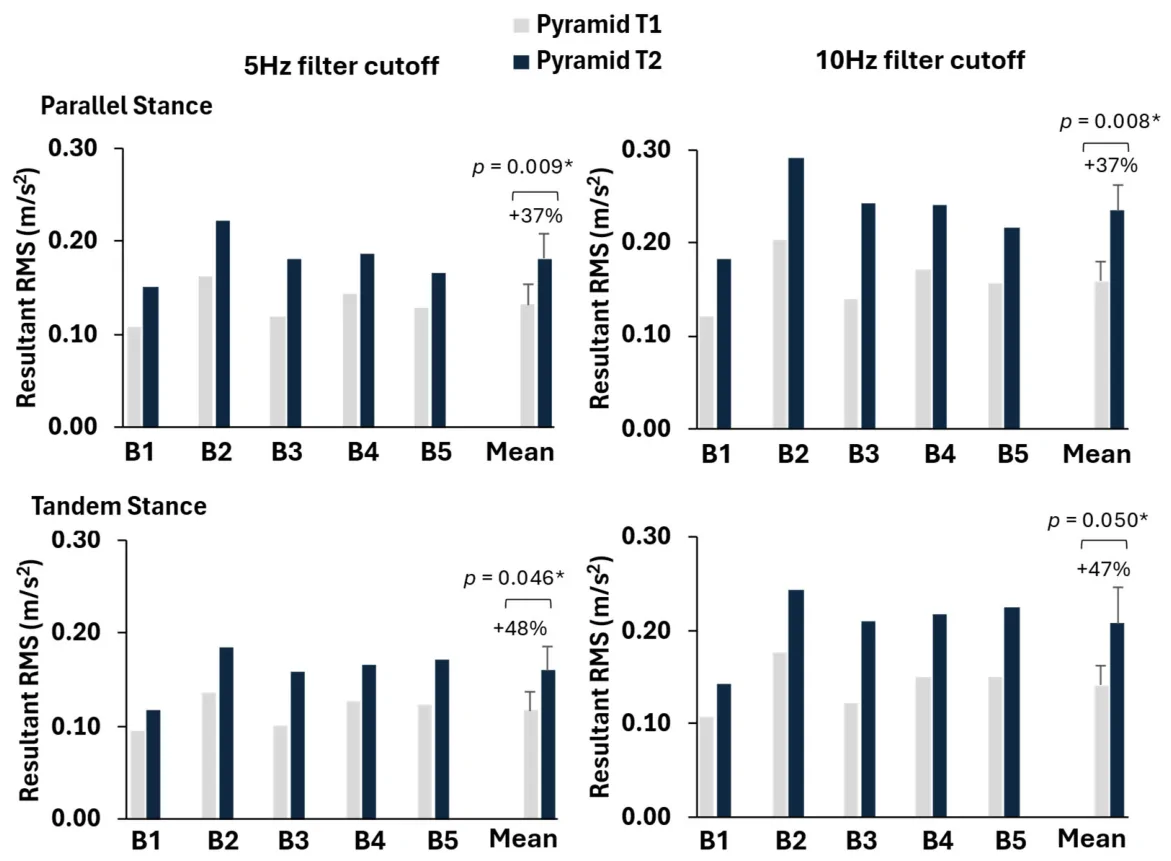
Individual resultant acceleration *RMS* of the five bases (B1, B2, B3, B4, B5), as well as their mean, across the parallel and tandem stances, in Pyramid T1 and Pyramid T2, at the 5 Hz (**left**) and 10 Hz (**right**) filter cutoff. The whiskers in the mean bars denote the respective standard deviation. * Significant base difference between the two pyramid configurations.

Regardless of the filter cutoff frequencies, the pyramid pairwise comparisons revealed significantly increased base-athlete resultant *RMS* acceleration in Pyramid T2, in both the parallel (+37% in both filter cutoffs) and the tandem (+48% and +47%, for the 5 Hz and the 10 Hz filter cutoffs, respectively) (Figure 5). As shown in Figure 5, all individual base-athletes increased their resultant *RMS* acceleration when supporting Top 2; however, the increase varied among them.

### 3.4. Main Effects—Interaction of Pyramid and Stance Configuration on Base’s Directionally Normalized Acceleration RMS

AP *RMS*_norm_. Repeated-measures analyses of AP *RMS*_norm_ revealed significant main effects of pyramid configuration at both 5 Hz (*F*(1,4) = 42.287, *p* = 0.003, partial *η*^2^*_p_* = 0.914, 90% CI [0.478, 0.947]) and 10 Hz (*F*(1,4) = 36.504, *p* = 0.004, partial *η*^2^*_p_* = 0.901, 90% CI [0.428, 0.939]), indicating a very large effect of top-athlete variations. Significant main effects were also observed for stance configuration at 5 Hz (*F*(1,4) = 26.246, *p* = 0.007, partial *η*^2^*_p_* = 0.868, 90% CI [0.317, 0.920]) and 10 Hz (*F*(1,4) = 32.296, *p* = 0.005, partial *η*^2^*_p_* = 0.890, 90% CI [0.387, 0.933]), demonstrating substantial differences between the parallel and tandem stance configurations.

Furthermore, the interaction between the pyramid and stance configurations reached statistical significance at both 5 Hz (*F*(1,4) = 11.860, *p* = 0.026, partial *η*^2^ = 0.748, 90% CI [0.085, 0.851]) and 10 Hz (*F*(1,4) = 8.839, *p* = 0.041, partial *η*^2^ = 0.688, 90% CI [0.024, 0.818]). The large effect sizes associated with these interactions (partial *η*^2^ > 0.68) highlight that the magnitude AP *RMS*_norm_ depended heavily on the specific combination of stance type and pyramid configuration.

**Figure 6:**
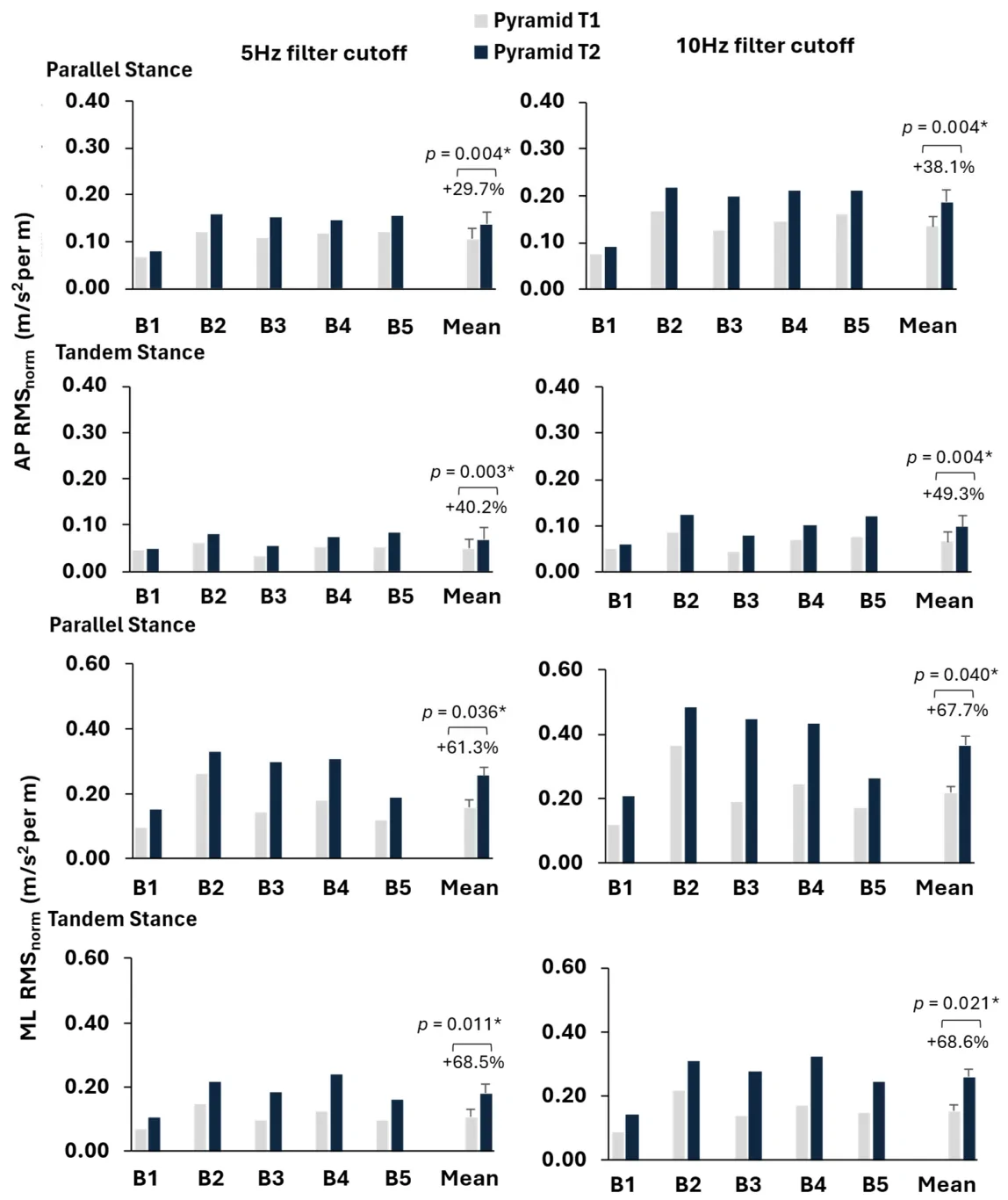
Normalized root mean square ($RMS_{\mathrm{norm}}$) of directionally normalized acceleration *RMS* (top: AP *RMS*_norm_, bottom: ML *RMS*_norm_ in the five base gymnasts (B1, B2, B3, B4, B5) and their group mean, across the parallel and tandem stances in Pyramid T1 (light gray bars) and Pyramid T2 (dark blue bars) for the 5 Hz (**left column**) and 10 Hz (**right column**) filter cutoffs. Whiskers on the mean bars denote standard deviations. Asterisks represent significant paired comparisons between Pyramid T1 and Pyramid T2 (p≤0.05), with corresponding percentage increases shown for the group means. Units are expressed in meters per second squared per meter of base envelope (${m/s}^{2}\;\mathrm{per}\;m$).

Regardless of the filter cutoff, pairwise comparisons revealed significantly increased AP *RMS*_norm_ in Pyramid T2 (with the increase being consistent across all individual bases) (Figure 6). For the parallel stance, Pyramid T2 increased AP *RMS*_norm_ by +29.7% at 5 Hz and +38.1% at 10 Hz (mean increase of 0.031 and 0.051, respectively). For the tandem stance, Pyramid T2 increased AP *RMS*_norm_ by +40.2% at 5 Hz and +49.3% at 10 Hz (mean increase of 0.020 and 0.032, respectively).

ML *RMS*_norm_. Repeated-measures analyses on the ML *RMS*_norm_ revealed significant large main effects for pyramid configuration at both 5 Hz *F*(1,4) = 16.624, *p* = 0.015, partial *η*^2^ = 0.806, 90% CI [0.173, 0.884]) and 10 Hz (*F*(1,4) = 13.303, *p* = 0.022, partial *η*^2^ = 0.769, 90% CI [0.113, 0.863]). Significant main effects were also observed for stance configuration (*F*(1,4) = 32.461, *p* = 0.005, partial *η*^2^ = 0.890, 90% CI [0.246, 0.943]) and 10 Hz (*F*(1,4) = 28.295, *p* = 0.006, partial *η*^2^ = 0.876, 90% CI [0.202, 0.935]), demonstrating substantial differences in ML *RMS*_norm_ acceleration between the parallel and tandem configurations.

Unlike AP *RMS*_norm_ acceleration, the interaction between pyramid and stance configurations did not reach statistical significance for ML *RMS*_norm_ acceleration at either 5 Hz (*F*(1,4) = 3.867, *p* = 0.121, partial *η*^2^*_p_* = 0.492, 90% CI [0.000, 0.751]) or 10 Hz (*F*(1,4) = 4.529, *p* = 0.100, partial *η*^2^*_p_* = 0.531, 90% CI [0.000, 0.769]). This finding indicates that the influence of the top-athlete variations on ML *RMS*_norm_ acceleration was similar across both stance types.

Regardless of the filter cutoff, pairwise comparisons revealed significantly increased ML *RMS*_norm_ in Pyramid T2 (with the increase being consistent across all individual bases) (Figure 6). For the parallel stance, Pyramid T2 increased ML *RMS*_norm_ by +61.3% at 5 Hz and +67.7% at 10 Hz (mean increase of 0.097 and 0.148, respectively). For the tandem stance, Pyramid T2 increased ML *RMS*_norm_ by +68.5% at 5 Hz and +68.6% at 10 Hz (mean increase of 0.074 and 0.105, respectively).

## 4. Discussion

The purpose of this study was to evaluate the postural stability and inter-base coupling mechanics of base gymnasts supporting distinct acrobatic pyramid configurations across different stance types and filtering thresholds. The primary findings revealed that pyramid configuration, naturally resulting from different top-athlete’s variations, heavily dictates postural stability: supporting Pyramid T2 produced significantly greater resultant and directionally normalized acceleration magnitudes than Pyramid T1, reflecting the performance demands and structural difficulty levels for competition frameworks.

Evaluating free floor-standing baselines alongside pyramid structures provides critical insight into the absolute mechanical burden imposed on base-athletes during partner integration. Under unconstrained floor conditions, bases displayed low, stable acceleration profiles (ranging from 0.0163 to 0.0265 m/s^2^), reflecting efficient baseline postural control in the absence of external perturbations. Structural integration into pyramid configurations disrupted this equilibrium, inducing substantial acceleration amplification across both stance geometries. In the parallel stance, supporting Pyramid T1 elevated base acceleration above floor baselines by a group mean of 37.5% (ranging from +28.1% to +58.5%) whereas supporting Pyramid T2 induced far greater perturbation, surging by a group mean of +67.3% (ranging from +51.8% to 74.8%). A similar pattern was observed in the tandem stance, where Pyramid T1 elevated base acceleration by +23.9% above floor baseline, compared to a +54.8% surge in Pyramid T2 (5 Hz). This baseline-to-pyramid comparison indicates that, in the base-athletes, the shank acceleration increased during pyramid execution, with larger increases observed in Pyramid T2 than in Pyramid T1. This pattern supports the view that the two top-athlete variations imposed different acceleration demands on the bases during partner-supported balance. Nevertheless, the contribution of specific top-athlete characteristics such as mass, physique, balancing ability, or force-transmission behavior cannot be isolated from the present accelerometry data.

The analysis demonstrated strong positive relationships in inter-base consistency across stance configurations, confirming that individual base gymnasts maintain stable relative performance hierarchies regardless of top-athlete support demands, extending similar previous findings [5,6,7]. Primary non-parametric evaluation confirmed statistically significant monotonic rank agreement across both stance types at the 5 Hz cutoff (*ρ* = 0.90, *p* = 0.037) and perfect rank order preservation in the tandem stance at 10 Hz (*ρ* = 1.00, *p* < 0.001).

Under parametric testing, linear correlations ranged from strong to near-significant thresholds (*r* = 0.86–0.92, *p* = 0.027–0.061), with high coefficients of determination (*R*^2^ = 0.74–0.85), denoting substantial shared variance and structural mechanical alignment. This divergence highlights how parametric linear associations can be slightly attenuated by small-sample degrees of freedom (N=5) and non-normal spacing artifacts, whereas non-parametric rank order remains robust. These findings align with established principles of load distribution, which suggest that consistent postural execution strategies are maintained even when external load demands vary [8,9]. Furthermore, these structural rank patterns remained invariant across both 5 Hz and 10 Hz low-pass filter cutoffs, confirming that wearable inertial sensors reliably capture body sway dynamics without distortion from filtering choices [19,20,21]. In contrast, base-to-top acceleration coupling exhibited wider variability and weaker correlations across most conditions (p>0.05), indicating that the accelerometric responses of the base- and top-athletes were not consistently mirrored in this exploratory sample. Rather than supporting a uniform base-to-top coupling pattern, these findings suggest that each base-top pairing may involve partner-specific acceleration adjustments during the shoulder-stand pyramid, consistent with previous work emphasizing inter-partner coordination and sensory–motor contributions to postural control [6,25].

Unnormalized acceleration metrics illustrated that structural complexity directly challenges base stability, matching core concepts of human balance control and external load management [24]. The substantial elevation in acceleration under Pyramid T2 conditions highlights the direct mechanical cost of supporting more demanding top geometries, which amplify inertial perturbations transmitted down the kinetic chain, consistent with findings on external load mass and placement [8,9]. Moreover, the main effects of stance configuration emphasize how geometric constraints dictate postural regulation. Constrained or narrower bases alter the body’s mechanical degrees of freedom, forcing distinct neuromuscular control strategies and altering baseline stability limits as a function of foot placement and load positioning [11,14]. The consistency of these findings across different filter frequencies further aligns with established frequency-domain methodologies for isolating low-frequency postural adjustments from movement artifacts [24].

Normalizing acceleration against physical stance-envelope dimensions provided biomechanical refinement, uncovering directional control mechanisms that raw metrics obscure. The significant interaction effects observed in AP *RMS*_norm_ acceleration demonstrate that sagittal plane stability is tightly coupled with stance-envelope dimensions, corroborating models of load distribution and directional foot placement [8,16]. Conversely, ML *RMS*_norm_ acceleration exhibited uniform main effects without interactions, indicating that frontal plane stabilization scales are proportionally relative to the stance-envelope width regardless of stance type, which mirrors multi-directional postural responses observed under staggered and asymmetric stance configurations [37,38]. The presence of large effect sizes (partial *η*^2^ > 0.76) confirms that spatial boundary scaling is crucial for evaluating true neuromuscular control strategies during multi-person athletic tasks [39,40].

A central and counter-intuitive finding of this work was the divergence between the top athletes’ isolated stability and the mechanical demands they impose on the bases. During independent free floor-standing baselines, Top 1 exhibited higher acceleration *RMS* (difference from 0.114 to 0.131 m/s^2^) compared to Top 2 (difference from 0.068 to 0.097 m/s^2^). Conventionally, one might expect that supporting a more stable, rigid individual (Top 2) would reduce the stabilizing burden on the base. However, the pyramid data revealed the exact opposite: supporting Top 2 induced significantly higher acceleration magnitudes and greater utilization of the bases’ spatial boundary constraints.

When integrated into the pyramid structure, both top athletes experienced a dramatic amplification in acceleration relative to their free floor baselines. Notably, Top 2’s acceleration surged by over 220% to 260% (reaching up to 0.327 m/s^2^) in the parallel 10 Hz cutoff, ultimately surpassing Top 1 during the parallel stance. This reversal highlights a crucial phenomenon in acrobatic biomechanics: a top athlete who appears steady in isolation may transmit unyielding, high-frequency perturbations down the kinetic chain. Conversely, a top athlete with slightly higher micro-movements on the floor (Top 1) may possess functional compliance or dynamic control strategies that smooth out load transfer, ultimately making them easier for the bases to stabilize.

## 5. Limitations

Several methodological limitations should be considered. First, the small sample size (*n* = 5) restricts statistical power and generalizability, increasing vulnerability to Type II errors despite large effect sizes. Acknowledging this limitation, the present study is explicitly presented as an exploratory one; however, it provides promise for recruiting larger cohorts of elite partnerships, expanding upon the limited normative data established for elite youth acrobatic populations [18]. Furthermore, the study implemented robust repeated-measures, counterbalanced design—where each base performed multiple trials across conditions—and prioritized magnitude-based inference and effect sizes alongside exact *p*-values, adhering to methodological standards established for specialized sports biomechanics research. Second, cross-sectional design captures static pyramids under controlled conditions, which may not fully replicate high-stress competitive environments. Furthermore, as the heavier top athlete also differed in age, height, and inherent postural stability, the top-athlete conditions completely confound mass with other physical and skill-related variables. Consequently, observed differences between the lighter-and the heavier-top conditions reflect an integrated response to the overall top-athlete physical and skill profile rather than an isolated effect of mass alone. Additionally, the prior joint training experience and familiarity varied across base-top pairings. In acrobatic gymnastics, inter-partner synergy and subconscious anticipatory adjustments develop over long-term shared practice; thus, unstandardized pairing history represents a potential confounding factor that may have influenced the individual dynamic stability strategies observed across conditions. Also, the stance area was calculated using a bounding rectangular envelope (length multiplied by width) rather than a complex anatomical polygon defined by the exact foot outlines. While this bounding-rectangle approach is necessary for orthogonal directional normalization (AP *RMS*_norm_ and ML *RMS*_norm_) it represents a standardized spatial boundary rather than the true anatomical surface area of the feet base of support. Finally, integration of surface electromyography and joint kinematic profiling would allow the usage of muscle co-contraction and trunk stiffness models for a deeper insight into how base gymnasts manage distinct loads [41,42,43].

## 6. Practical Applications

The findings of this exploratory study contribute to biomechanical research in acrobatic gymnastics [39] and suggest that wearable inertial sensors may provide practitioners with descriptive information on how base-athlete shank acceleration changes across specific top-athlete variations and stance configurations in acrobatic shoulder-stand pyramids. Directionally normalized acceleration metrics, such as AP *RMS*_norm_ and ML *RMS*_norm_, may help characterize whether acceleration responses differ more prominently in the anteroposterior or mediolateral direction relative to the available stance-envelope dimensions.

## 7. Conclusions

This exploratory study showed the acceleration demands experienced by base gymnasts during acrobatic shoulder-stand pyramids, demonstrating that postural stability varies across the two specific top-athlete variations examined, (lighter-top versus heavier-top) and base foot placement configurations (parallel versus tandem). Utilizing wearable inertial sensors alongside normalization against the stance-envelope dimensions revealed that structural complexity (Pyramid T1 versus Pyramid T2) significantly increases acceleration magnitudes, particularly in the AP direction, while highlighting consistent inter-base performance hierarchies. Although limited by a small sample size and the inherent confounding of top-athlete characteristics, these findings underscore the necessity of accounting for spatial boundaries and structural loading when evaluating postural control in multi-person athletic tasks. Future research could elaborate on the role of top-athlete’s mass or physique on postural stability during a variety of acrobatic balance tasks.

## Figures and Tables

**Table 1:** Mean values ± standard deviation of participants’ characteristics.

	Age (yrs)	Body Mass (kg)	Body Height (m)	Body Mass Index (kg/m^2^)
Top 1	7.7	22.7	1.22	15.3
Top 2 *	10.7	36.2	1.46	17.0
Base 1	15.7	60.1	1.65	22.1
Base 2	13.9	53.0	1.66	19.2
Base 3	14.8	51.2	1.58	20.5
Base 4	14.0	50.7	1.58	20.3
Base 5	13.5	62.7	1.65	23.0
Mean ± SD Tops	9.2 ± 2.1	29.5 ± 9.5	1.34 ± 0.17	16.1 ± 1.2
Mean ± SD Bases	14.4 ± 0.9	55.5 ± 5.5	1.62 ± 0.04	21.0 ± 1.5

* Top 2 is 13.5 kg (+59%) heavier and by 0.24 m (+20%) taller than Top 1.

**Table 2:** Length, width and area of the bounding-rectangle stance-envelope in the parallel and tandem configurations for each one of the five base-athletes, as well as their mean, standard deviation (SD) and relative coefficient of variation (CV) expressed as percentage (%). The percentage difference of the tandem compared to the parallel stance is also presented.

	Parallel Stance			Tandem Stance			Percentage (%) Difference Tandem to Parallel (100%) Stance		
	Length (cm)	Width (cm)	Stance-Envelope Area (cm^2^)	Length (cm)	Width (cm)	Stance-Envelope Area (cm^2^)	Length (cm)	Width (cm)	Stance- Envelope Area (cm^2^)
Base 1	25.5	30.5	777.8	43.0	40.0	1720.0	+68.6%	+22.1%	121.2%
Base 2	24.5	28.0	686.0	46.0	41.5	1909.0	+87.8%	+29.3%	178.3%
Base 3	23.5	29.5	693.2	50.0	43.0	2150.0	+112.8%	+27.0%	210.1%
Base 4	23.5	28.0	658.0	38.5	36.5	1405.2	+63.8%	+22.1%	113.6%
Base 5	24.0	34.0	816.0	41.0	43.5	1783.5	+70.8%	+23.2%	118.6%
Mean	24.2	30.0	726.2	43.7	40.9	1793.6	+80.8%	+24.7%	+148.3%
SD	0.75	2.21	67.2	3.99	2.52	272.3	+20.1% units	+3.2% units	+43.4% units
CV%	3.5%	8.2%	9.3%	10.2%	6.9%	15.2%	24.8%	13.1%	29.3%

**Table 3:** Base-athletes’ mean ± SD Resultant acceleration *RMS* (m/s^2^) as well as mean difference (standard error), Bonferroni-adjusted *p* values (*p*_adj_) and 95% CI (Confidence Intervals) for the pairwise comparisons, across free floor standing and pyramid variations (Pyramid T1, Pyramid T2), in the parallel and tandem stance at 5 Hz and 10 Hz filter cutoffs.

Filter Cutoff	Condition/Stance	Mean ± SD (m/s^2^)	Mean ^a^ (Standard Error) of Difference ^a^	*p*_adj_	95% CI for Difference
Parallel Stance					
5 Hz	Free floor standing	0.095 ± 0.008	non applicable		
	Pyramid T1	0.132 ± 0.021	0.026 (0.004)	*p*_adj_ < 0.001 *	[0.017, 0.036]
	Pyramid T2	0.181 ± 0.027	0.058 (0.007)	*p*_adj_ < 0.001 *	[0.039, 0.077]
10 Hz	Free floor standing	0.101 ± 0.011	non applicable		
	Pyramid T1	0.159 ± 0.032	0.039 (0.005)	*p*_adj_ < 0.001 *	[ 0.024, 0.053]
	Pyramid T2	0.235 ± 0.040	0.087 (0.011)	*p*_adj_ < 0.001 *	[0.059, 0.116]
Tandem Stance					
5 Hz	Free floor standing	0.085 ± 0.011	non applicable		
	Pyramid T1	0.117 ± 0.018	0.023 (0.003)	*p*_adj_ < 0.001 *	[0.015, 0.030]
	Pyramid T2	0.160 ± 0.025	0.051 (0.006)	*p*_adj_ < 0.001 *	[0.035, 0.067]
10 Hz	Free floor standing	0.093 ± 0.017	non applicable		
	Pyramid T1	0.142 ± 0.027	0.034 (0.004)	*p*_adj_ < 0.001 *	[0.022, 0.045]
	Pyramid T2	0.208 ± 0.038	0.077 (0.009)	*p*_adj_ < 0.001 *	[0.053, 0.100]

Based on estimated marginal means. ^a^ Values represent the positive increase from free floor standing to pyramid variations. Adjustment for multiple comparisons: Bonferroni. * Significance at 0.05 level.

**Table 4:** Spearman rank correlation (ρ), Pearson correlation (r), coefficient of determination ($R^{2}$), and exact p-values across stance configurations and filter cutoffs (N=5).

Stance	Comparison	Spearman *ρ*	Pearson *r*, *R*^2^
5 Hz filter cutoff			
Parallel	Base, Pyramid T1 vs. Pyramid T2	*ρ* = 0.900, *p* = 0.037 *	*r* = 0.920, *R*^2^ = 0.850, *p* = 0.027 *
	Pyramid T1, Base vs. Top	*ρ* = 0.300, *p* = 0.624	*r* = 0.170, *R*^2^ = 0.030, *p* = 0.790
	Pyramid T2, Base vs. Top	*ρ* = 0.900, *p* = 0.037 *	*r* = 0.780, *R*^2^ = 0.610, *p* = 0.118
Tandem	Base, Pyramid T1 vs. Pyramid T2	*ρ* = 0.900, *p* = 0.037 *	*r* = 0.860, *R*^2^ = 0.740, *p* = 0.061
	Pyramid T1, Base vs. Top	*ρ* = −0.300, *p* = 0.624	*r* = −0.250, *R*^2^ = 0.060, *p* = 0.689
	Pyramid T2, Base vs. Top	*ρ* = 0.100, *p* = 0.873	*r* = 0.510, *R*^2^ = 0.260, *p* = 0.383
10 Hz filter cutoff			
Parallel	Base, Pyramid T1 vs. Pyramid T2	*ρ* = 0.700, *p* = 0.188	*r* = 0.890, *R*^2^ = 0.780, *p* = 0.046 *
	Pyramid T1, Base vs. Top	*ρ* = 0.500, *p* = 0.391	*r* = 0.410, *R*^2^ = 0.170, *p* = 0.492
	Pyramid T2, Base vs. Top	*ρ* = 0.700, *p* = 0.188	*r* = 0.600, *R*^2^ = 0.360, *p* = 0.284
Tandem	Base, Pyramid T1 vs. Pyramid T2	*ρ* = 1.000, *p* < 0.001 *	*r* = 0.870, *R*^2^ = 0.750, *p* = 0.058
	Pyramid T1, Base vs. Top	*ρ* = 0.300, *p* = 0.624	*r* = −0.060, *R*^2^ = 0.003, *p* = 0.926
	Pyramid T2, Base vs. Top	*ρ* = 0.200, *p* = 0.747	*r* = 0.390, *R*^2^ = 0.150, *p* = 0.516

* Statistical significance at *p* ≤ 0.05. Spearman effect size (|ρ|) [35]: 0.00–0.29: negligible/weak association, 0.30–0.49: moderate association, 0.50–0.69: strong association, 0.70–0.99: very strong association, 1.00: perfect monotonic relationship. Pearson effect size: *r* = 0.10 small effect, *r* = 0.30 medium effect, and *r* = 0.50 large effect [36].

## Data Availability

The original contributions presented in this study are included in the article/Supplementary Material. Further inquiries can be directed to the corresponding author.

## Supplementary Materials

The following supporting information can be downloaded at: https://www.mdpi.com/article/10.3390/s26185831/s1, Supplementary File S1. MATLAB script for calculating non-centrality parameter-based confidence intervals for partial *η*^2^ effect sizes.
