# Supplementary material for: An Exploratory Analysis of Postural Stability in Acrobatic Shoulder-Stand Pyramids Using Inertial Sensors: The Interplay of Top-Athlete’s Variations and Base-Athlete’s Stance Configurations
Source: Sensors (Basel). 2026 Sep 14;26(18):5831. doi: 10.3390/s26185831 (PMC13611068; doi:10.3390/s26185831)
Supplement: Supplementary file 1 [file sensors-26-05831-s001.zip › sensors-4469060-supplementary.pdf]

Supplementary Material

Supplementary File S1. MATLAB script for calculating non-centrality parameter-based confidence intervals for partial η^2 effect sizes.

```
% Inputs from your ANOVA
F =………; % write the F value
df1 = 1; % Numerator degrees of freedom
df2 = 4; % Denominator degrees of freedom
conf_level = 0.95; % Standard 90% CI corresponding to alpha = .05 for squared effect sizes

alpha = 1 - conf_level;

% Solve for the lower and upper non-centrality parameter (lambda) bounds
fun_lower = @(ncp) ncfcdf(F, df1, df2, ncp) - (1 - alpha / 2);
fun_upper = @(ncp) ncfcdf(F, df1, df2, ncp) - (alpha / 2);

try
   lambda_lower = fzero(fun_lower, [0, 1000]);
catch
   lambda_lower = 0; % Defaults to 0 when the lower bound collapses for small F values
end
lambda_upper = fzero(fun_upper, [0, 1000]);

% Convert lambda bounds to partial eta-squared bounds
eta_lower = lambda_lower / (lambda_lower + df1 + df2 + 1);
eta_upper = lambda_upper / (lambda_upper + df1 + df2 + 1);

fprintf('90%% CI: [%.3f, %.3f]\n', eta_lower, eta_upper);
```
